# Drought-Tolerant *Brassica rapa* Shows Rapid Expression of Gene Networks for General Stress Responses and Programmed Cell Death Under Simulated Drought Stress

**DOI:** 10.1007/s11105-017-1032-4

**Published:** 2017-05-22

**Authors:** Yi Ming Guo, Birgit Samans, Sheng Chen, Kidist B. Kibret, Sarah Hatzig, Neil C. Turner, Matthew N. Nelson, Wallace A. Cowling, Rod J. Snowdon

**Affiliations:** 1 UWA School of Agriculture and Environment, The University of Western Australia, Perth, 6009 Australia; 2 The UWA Institute of Agriculture, The University of Western Australia, Perth, 6009 Australia; 3 Crop Research Institute, Hunan Academy of Agricultural Sciences, Changsha, China; 4 Department of Plant Breeding, Justus Liebig University, 35392 Giessen, Germany; 5 Centre for Plant Genetics and Breeding, The University of Western Australia, Perth, 6009 Australia; 6 Natural Capital and Plant Health, Royal Botanic Gardens Kew, Wakehurst Place, Ardingly, West Sussex RH17 6TN UK

**Keywords:** RNA sequencing, Abscisic acid, Salicylic acid, Jasmonic acid, Gene ontology, Enrichment analysis

## Abstract

Production of oilseed rape/canola (*Brassica napus*) is increasingly threatened by dry conditions while the demand for vegetable oil is increasing. *Brassica rapa* is a genetically diverse ancestor of *B. napus*, and is readily crossed with *B. napus*. Recently, we reported promising levels of drought tolerance in a wild type of *B. rapa* which could be a source of drought tolerance for *B. napus*. We analysed global gene expression by messenger RNA sequencing in seedlings of the drought-tolerant and a drought-sensitive genotype of *B. rapa* under simulated drought stress and control conditions. A subset of stress-response genes were validated by reverse transcription quantitative PCR. Gene ontology enrichment analysis and pathway enrichment analysis revealed major differences between the two genotypes in the mode and onset of stress responses in the first 12 h of treatment. Drought-tolerant plants reacted uniquely and rapidly by upregulating genes associated with jasmonic acid and salicylic acid metabolism, as well as genes known to cause endoplasmic reticulum stress and induction of programmed cell death. Conversely, active responses in drought-sensitive plants were delayed until 8 or 12 h after stress application. The results may help to identify biomarkers for selection of breeding materials with potentially improved drought tolerance.

**Electronic supplementary material:**

The online version of this article (doi:10.1007/s11105-017-1032-4) contains supplementary material, which is available to authorized users.

## Introduction

Meeting growing global demand for vegetable oil is becoming increasingly challenging in the face of climate change, with drought conditions threatening oilseed production in many parts of the world (Tester and Langridge 2010). The allopolyploid oilseed crop *Brassica napus* L. (canola, rapeseed, oilseed rape; genome AACC, 2n = 38) is the most important *Brassica* species worldwide (Prakash et al. 2012) and is the second-most important oilseed crop in temperate climatic areas. However, low genetic diversity (Becker et al. 1995; Bus et al. 2011; Cowling 2007) in this recently evolved species limits the possibility to breed for drought tolerance and other environmental adaptation traits, meaning that identification of variation for drought-tolerance genes from related species has become urgent for canola breeding. In contrast, *Brassica rapa* L. (AA, 2n = 20), one of the diploid ancestor species of *B. napus*, is renowned for its wide genetic diversity (Annisa and Cowling 2013; Guo et al. 2014; Ofori et al. 2008; Zhao et al. 2005). Interspecific crosses between these *B. rapa* and *B. napus* are relatively easy to achieve, so there is great potential to transfer useful genetic variation from *B. rapa* into *B. napus*.

Plants have evolved various mechanisms to adapt to and cope with water stress conditions by implementing a series of biochemical, molecular, physiological and/or morphological changes (Moore et al. 2009; Turner 1997). Over the last decade, efforts have focussed on understanding these complex mechanisms and recent progress has been extensively reviewed (Cattivelli et al. 2008; Huang et al. 2008; Varshney et al. 2011). Recently, gene expression profiling techniques based on ultra-high throughput messenger RNA sequencing (mRNA-Seq) have brought unprecedented resolution to global transcription profiling including the potential for discovery of new transcripts or genes (Ozsolak and Milos 2011; Wang et al. 2009). The whole genome sequences of *B. rapa* (Wang et al. 2011) and *B. napus* (Chalhoub et al. 2014) enabled direct associations of transcriptome sequence data with *Brassica* crop genomes (Tong et al. 2013). Comparisons of transcriptomes under stressed vs. control conditions can help to gain a comprehensive insight into how plants respond to stresses and to identify and characterize the genes and gene networks responsible for mounting stress tolerance responses. For example, Yu et al. (2012) and Liu et al. (2015) identified a large number of differentially expressed genes after dehydration stress in Chinese cabbage and *B. napus*, respectively, revealing a high transcription complexity involved in this process. Therefore, identification of differences in transcriptional responses between drought-sensitive and drought-tolerant *B. rapa* accessions in response to osmotic stress can potentially provide new information about the mechanisms and regulation of stress tolerance, and help identify novel methods to breed for drought tolerance.

An essential step for transcriptome analysis is the biological interpretation of observed differences in gene expression. Gene ontology (GO) analyses provide an approach to associate transcriptome sequence data with biological understanding by linking differentially expressed genes to putative molecular functions (Falcon and Gentleman 2007). However, genes showing significant responsiveness to stress are not necessarily the most effective targets for enhanced drought tolerance and yield protection (Serraj and Sinclair 2002). Rather, the pattern of significant biological or molecular changes arising from a group of genes is of more interest, because these describe the general response mechanisms associated with the stress tolerance phenotype.

A relatively small number of well-known hormones and transcription factors are actively involved with regulating tolerance to stresses, such as abscisic acid (ABA), salicylic acid (SA), jasmonic acid (JA) and calcium-mediated transcription factors (Shinozaki and Yamaguchi-Shinozaki 2007). Among the regulatory metabolites driving drought responses, the phytohormone ABA is arguably the most important. ABA controls the expression of drought stress-related genes and is responsible for closure of the stomatal aperture (Cutler et al. 2010; Davies and Zhang 1991). Some links have been demonstrated between ABA function and sulphur metabolism. For example, in maize (*Zea mays*) sulphate was found to be the only xylem-borne metabolite that follows ABA transport from root to shoot and regulates the ABA-induced closure of stomata in the leaves (Ernst et al. 2010). Glucosinolates, a group of sulphur-rich compounds found exclusively in the Brassicaceae, are also believed to be involved in the plant defence system and possibly with abiotic stress tolerance (Halkier and Gershenzon 2006). Whereas glucosinolate contents in seeds of canola-quality *B. napus* have been largely reduced by intensive breeding for seed meal quality (Snowdon and Friedt 2004), an enormous diversity for glucosinolate compounds is found in leaves of different *B. rapa* accessions (Cartea and Velasco 2008) with various pathways (Pino Del Carpio et al. 2014). Variation in glucosinolate synthesis and its response to stress may be of particular interest as a potential factor contributing to drought tolerance in *Brassica* species.

Apart from metabolite accumulation during osmotic stress, programmed cell death (PCD) is another well-known factor in plant defence (van Doorn and Woltering 2004) and disease susceptibility (Li et al. 2008). PCD is an active cellular process that facilitates the removal of unwanted or damaged cells and is essential for cellular differentiation and tissue homeostasis (van Doorn et al. 2011). High salinity leads to ionic, osmotic and oxidative stress in plants, resulting in the induction of signalling events that lead to PCD in higher plants (Wang et al. 2010). Duan et al. (2010) reported the pattern and morphological characteristics of PCD induced by water stress, which might represent an important mechanism of plant drought tolerance at the organ level. It has also been demonstrated that PCD can be mediated by stress induced by caspase-like enzymatic activities in the endoplasmic reticulum (Cai et al. 2014).

In previous work, we identified considerable variation for drought tolerance among nine *B. rapa* and one *Brassica juncea* accessions that were subjected to detailed physiological evaluations of drought responses under controlled environment conditions (Guo et al. 2015b). A wild-type *B. rapa* ssp. *sylvestris* was found to maintain mature plant biomass following a transient drought stress during the early reproductive stage, compared to control conditions, whereas a Yellow Sarson type (*B. rapa* ssp. *triloculoris*) suffered significant reductions in mature plant biomass under the same conditions (Guo et al. 2015b). Similarly, in *B. napus*, we demonstrated that drought-tolerant and drought-susceptible cultivars exhibit differential physiological responses to osmotic stress, including considerable differences in ABA responses and osmotic adjustment (Hatzig et al. 2014). However, the genetic mechanisms underlying differences between drought-sensitive (DS) and drought-tolerant (DT) accessions remain unknown. Identification of the major drought response pathways and potential regulatory factors whose expression changes are in associated with drought-tolerant traits would be highly valuable for breeding of new drought-tolerant varieties.

The objectives of this study were (i) to assess genes and gene pathways in DT and DS *B. rapa* accessions which respond to osmotic stress vs. non-stress treatments; (ii) to elucidate differences between DT and DS accessions in temporal patterns of gene and network expression after stress application; and (iii) to investigate patterns of enrichment for GO and metabolic regulation pathways associated with response to drought in the DT and DS *B. rapa*.

## Materials and Methods

### Plant Materials and Growth Conditions

Two accessions of *B. rapa* (CR2355 and ATC92037) were selected as contrasting genotypes for this study. CR2355 is a wild-type *B. rapa* ssp. *sylvestris*, and was defined as drought tolerant when it maintained mature plant biomass following exposure to transient drought stress at the reproductive stage (Guo et al. 2015b). On the other hand, the Yellow Sarson type ATC92037 suffered significant biomass reduction in mature plants under the same drought stress treatment and is therefore considered to be drought sensitive (Guo et al. 2013; Guo et al. 2015b).

Plant materials were grown in a hydroponic growth system which enables controlled application of osmotic stress to the roots by addition of polyethylene glycol (PEG) 6000 (Carl Roth GmbH + Co. KG, Karlsruhe, Germany), combined with the ability to harvest not only leaf materials but also clean, soil-free root samples for gene expression profiling. The plant growth system and stress experiments have been previously described in detail by Hatzig et al. (2014).

Ninety-six seeds per genotype were germinated in 1% agar gel in plastic tubes (0.2 ml volume) at 10 °C in the dark for 2–4 days before removal of the bottom of the tubes and each transferred into a hole bored in the lid of a 50-ml Falcon tube containing Murashige and Skoog (MS) medium solution (Murashige and Skoog 1962) (Duchefa Biochemie, Haarlem, The Netherlands). After 7 days, the seedlings were transferred inside the lids of the tubes to open tubes inserted into 150 mm deep 10 L aquaria in a controlled environment chamber with a day length of 16 h, 60% humidity, day/night temperatures of 16 °C/12 °C and photosynthetically active radiation of 216–257 μmol m^−2^ s^−1^. The nutrient solution was changed at 7-day intervals.

At 25–27 days after sowing, when the plants had four to five true leaves (BBCH 14–15) (Lancashire et al. 1991), osmotic stress was applied to half of the seedlings by adding 2.5% PEG 6000 and the other half remained with MS medium as a control. The corresponding osmotic potentials of the different PEG solutions can be calculated as follows using the formula of Michel and Kaufmann (1973): −0.022 MPa at 2.5% PEG 6000. To ensure oxygen supply, the solutions were aerated using a pressure air pump.

### Tissue Sampling, Total RNA Extraction and Pooling

Whole shoots and roots were sampled 4, 8 and 12 h after imposition of the stress, shock-frozen immediately in liquid nitrogen and stored at −80 °C until RNA extraction. Three biological replicates per time point, genotype and stress treatment were harvested.

**Fig. 1:**
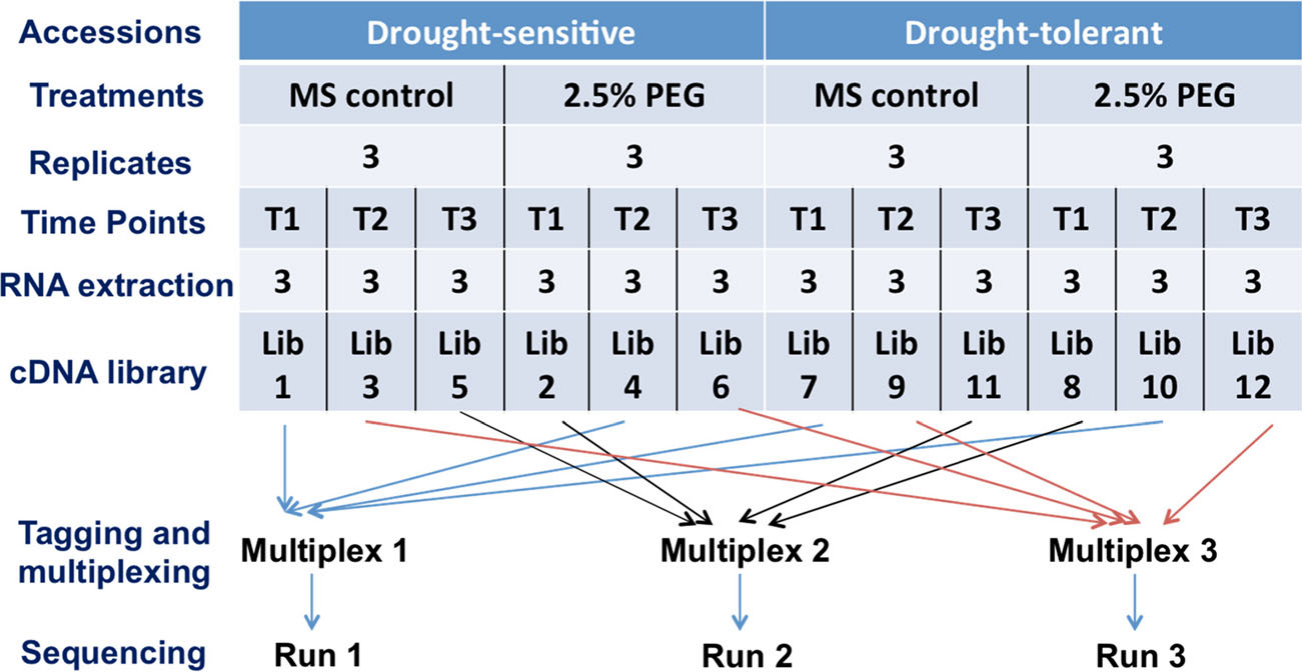
Research workflow employed in this experiment. The drought-sensitive and drought-tolerant genotypes of *Brassica rapa* were subjected to two treatments (*2.5% PEG* osmotic stress and *MS* medium control). Leaf tissues were sampled 4, 8 and 12 h after imposition of treatment, indicated by time points *T1*, *T2* and *T3*, respectively. Each library contained three biological replicates which were pooled into three multiplex for three sequencing runs

Total RNA was extracted using the RNEasy Mini Kit (Qiagen, Hilden, Germany) from shoot samples and treated with DNase using the RNase-free DNase Set (Qiagen, Hilden, Germany) during the process. The quantity and quality of all RNA samples were checked using a Fragment Analyzer™ Automated Capillary Electrophoresis system (Advanced Analytical, Heidelberg, Germany). Equimolar RNA samples from the three biological replicates were then pooled (Fig. 1) and used to prepare 12 complementary DNA (cDNA) libraries for mRNAseq analysis (2 genotypes × 3 time points × 2 stress treatments).

### cDNA Library Construction and Sequencing

cDNA libraries were prepared with the TruSeq Stranded Total RNA with Ribo-Zero™ Plant kit from Illumina (Illumina, San Diego, CA, USA), according to the manufacturer’s protocol. Each cDNA library was tagged with Multiplex Identifier barcode adaptors, with three independent multiplexes created by following the rule of randomizing (Fang and Cui 2011) with mixed libraries from different sampling time points (Fig. 1).

The quantity of each cDNA library was checked using the KAPA library quantification kit (KAPA Biosystems, Wilmington, MA, USA), and the fragment size was check by capillary electrophoresis using a QIAxcel Advanced (Qiagen, Hilden, Germany) before final multiplexing for sequencing. Each multiplex was freshly prepared and diluted as instructed by the MiSeq user manual (Illumina, San Diego, CA, USA). MiSeq reagent kits v2 (2 × 150 bp) containing pre-filled, ready-to-use cartridges were used for each sequencing run.

### Sequencing Data Analysis

#### Pre-processing and Mapping of Reads

After separation of the pooled raw sequence data according to the library-specific barcodes, read qualities of the sequences from the 12 mRNA-Seq libraries were initially checked using FastQC (http://www.bioinformatics.babraham.ac.uk/projects/fastqc/), and pre-processed by removing putative adapter sequences with the cutadapt package (Martin 2011), trimming the first 11 bp (to remove hexamer bias) and removing low-quality reads using the fastx_trimmer and fastq_quality_filter (-q 28 -p 95) algorithms from the fastx_toolkit (http://hannonlab.cshl.edu/fastx_toolkit/).

After pre-processing, the reads were aligned to the *B. rapa* cv. Chiifu reference genome v1.5 (Wang et al. 2011) allowing three mismatches, then normalized and quantified as fragments per kilo bases of exons for per million mapped reads (FPKM) values (fragments per kilo bases of exons for per million mapped reads) using the spliced aligner TopHat/Cufflinks (Trapnell et al. 2009; Trapnell et al. 2012).

#### Comparison of Differential Gene Expression

**Fig. 2:**
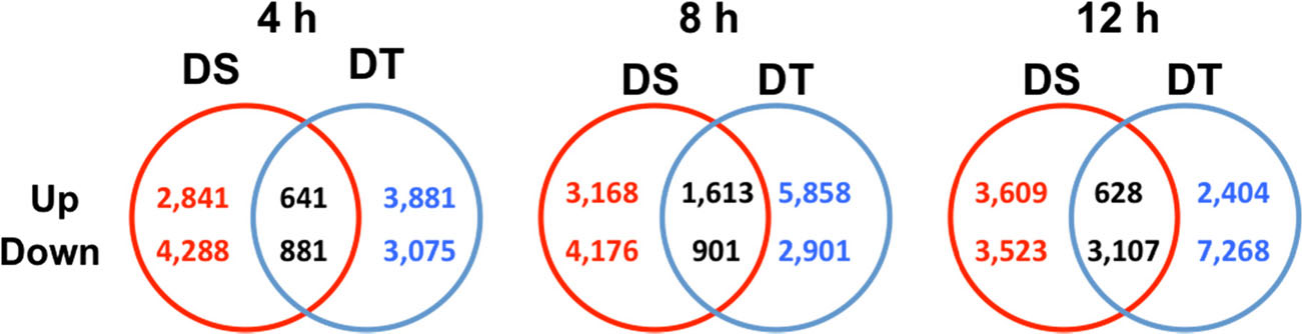
Venn diagrams showing the numbers of up- and down-regulated genes unique to either the drought-sensitive (DS) or the drought-tolerant (DT) *Brassica rapa* genotype or common to both genotypes at 4, 8 and 12 h after imposition of treatment

For both the DS and DT genotypes, the log_2_ ratios of the FPKM values were calculated over all three time points to compare the osmotically stressed (PEG) plants and the MS control samples. Differentially, expressed genes were filtered by a minimum fold change of 2. For each time point, genes differentially expressed in both genotypes or uniquely in one of the genotypes were obtained using Venn diagrams (Fig. 2). The original gene expression profiling data are available (Guo et al. 2015a).

#### Gene Ontology Enrichment Analysis

The sequences of the *B. rapa* transcriptome (Brassica_rapa_v1.5) were functionally annotated using Blast2GO (Conesa et al. 2005). The GO terms of differentially expressed genes were summarized and plotted using the online tool WEGO (Ye et al. 2006). The number of differentially expressed genes in different categories describing the biological process, molecular function or cellular components, respectively, was obtained from the WEGO summary output.

Genes commonly (in both genotypes) or uniquely (in one of the genotypes) upregulated when comparing PEG and control treatments were analysed for enriched GO terms at each time point using the GOstats package implemented within the R/bioconductor environment (Falcon and Gentleman 2007). Enriched GO terms and the corresponding *P* values (*P <* 0.01) were then used as input for the REViGO analysis (http://revigo.irb.hr) to reduce redundancy within lists of GO terms and visualize semantic clustering of the identified top scoring GO terms (sorted by *P* values) (Supek et al. 2011) (Table S1). A medium-sized similarity using the *Arabidopsis thaliana* database for GO terms was applied when processing the REViGO analysis.

#### Pathway Enrichment Analysis

Additionally, the gene lists with commonly and uniquely upregulated genes were analysed for enriched pathways. Pathway annotations for *B. rapa* were downloaded from the MapMan Store (http://mapman.gabipd.org/web/guest/mapmanstore), and for each pathway, an enrichment analysis was conducted to detect significantly enriched pathways with a Fisher’s exact test (*P* < 0.05) based on the *B. rapa* transcriptome as the background set (Table S2).

#### Heat Map Plotting and Dendrogram

Genes with documented responsiveness to drought or osmotic stress were selected from the GO annotation ‘responsive to stimulus’ to compare the gene expression changes. The ‘pheatmap’ package implemented in *R* (Kolde 2013) was used to generate the heat map based on the hierarchical clustering result.

### qRT-PCR

Plants of DS and DT were grown in steam-sterilized potting mix in 12-cm pots, and soil moisture was adjusted to field capacity on a daily basis. At first flower, water was withheld from the drought treatment for 7 days, but watering continued in the well-watered treatment. On the seventh day, poly-A+ RNA was extracted from leaves of the DT and DS accessions in both the drought and well-watered treatments using oligo-dT magnetic beads (Dynabeads, Invitrogen Life Technologies, Grand Island, NY). Single-strand cDNA was synthesized using Bioscript RT (Bioline, Alexandria, NSW) on the beads (Jost et al. 2007). Gene-specific primers for candidate stress-related genes found in the *B. rapa* reference genome sequence (Wang et al. 2011) were designed based on the nucleotide sequence of the chosen unigenes using Primer 3.0 Software (Rozen and Skaletsky 2000). Primers used in qRT-PCR analyses are summarized in Table S3. qRT-PCR reactions were run on the 7500FAST Sequence Detection System (Applied Biosystems, Life Technologies) using Power SYBR Green Master Mix (Applied Biosystems, Life Technologies). Reactions (10 μl) were carried out in 96-well format and contained approximately 0.5 ng cDNA, 2.5 μl of a mixture containing 1.2 μM each of the forward and reverse primers and 5 μl of master mix. The cycle threshold (Ct) and normalized fluorescence values were determined for each sample by using Prism Sequence Detector Software v. 2.0 (Applied Biosystems, Life Technologies) based on three biological replicates each with two technical replicates. Relative expression levels of target genes were calculated using the ∆∆Ct method (Livak and Schmittgen 2001) and with the housekeeping genes actin and EF1α mRNA as internal standards.

## Results

### Generation, Mapping and Assessment of mRNA-Seq Reads

Three MiSeq sequencing runs, sequencing 12 libraries, generated a total of 26,703,167 reads, with 25,434,703 reads (95.2%) passing Illumina’s internal filter criteria (PF reads). Of the PF reads, 25,153,104 (98.9%) unique reads were identified and used to proceed with FastQC quality checks. A total of 22,001,458 reads passed the quality checks and were mapped onto the *B. rapa* reference genome using TopHat, which applies the Bowtie2 algorithm for mapping (Table S1a). A total of 29,178 differentially expressed unigenes were identified across the 12 libraries (Table S1b).

### Differentially Expressed Genes in the DT and DS Accession

A total of 25,462 genes were identified as being differentially expressed between PEG-treated and MS control treatment over the three time points. The majority of differentially expressed genes were unique to either the DS or the DT accession; for example, at 4 h, 7129 genes were differentially expressed uniquely in the DS accession, 6956 genes were differentially expressed uniquely in the DT accession and 1522 differentially expressed genes were common to both accessions (Fig. 2). We focussed on genes and gene pathways that were unique to the DT or DS accession.

### Validation of RNA-Seq Data by qRT-PCR

A total of 29 candidate abiotic stress-related genes with low to high expression levels based on their RNA-Seq data after 12 h PEG treatment at the seedling stage were chosen for qRT-PCR analysis at first flower. Fold changes in RNA-Seq for 18 genes were validated by qRT-PCR (Table 1). Among these 18 genes, 4 genes were upregulated in both DS and DT, 4 genes were downregulated in both DS and DT and ten genes were upregulated in DS and downregulated in DT. For the remaining 11 of the 29 genes, the fold changes were consistent in the DS or the DT accession, but not in both accessions.

**Table 1:**
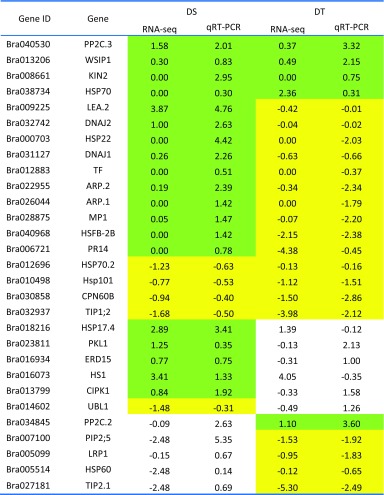
Comparison between RNA-Seq of 29 stress-related genes differentially expressed between PEG-treated and MS control treatment at 12 h and qRT-PCR at early flowering stage in drought-sensitive (DS) and drought-tolerant (DT) genotypes of *Brassica rapa*

Changes in fragments per kilo bases of exons for per million mapped reads (FPKM) of transcripts were calculated from RNA-Seq data and compared to log_2_-fold changes measured by qRT-PCR. Green and yellow shading represents consistent up- and downregulation, respectively, of genes in both approaches

### Gene Ontology Classification

**Fig. 3:**
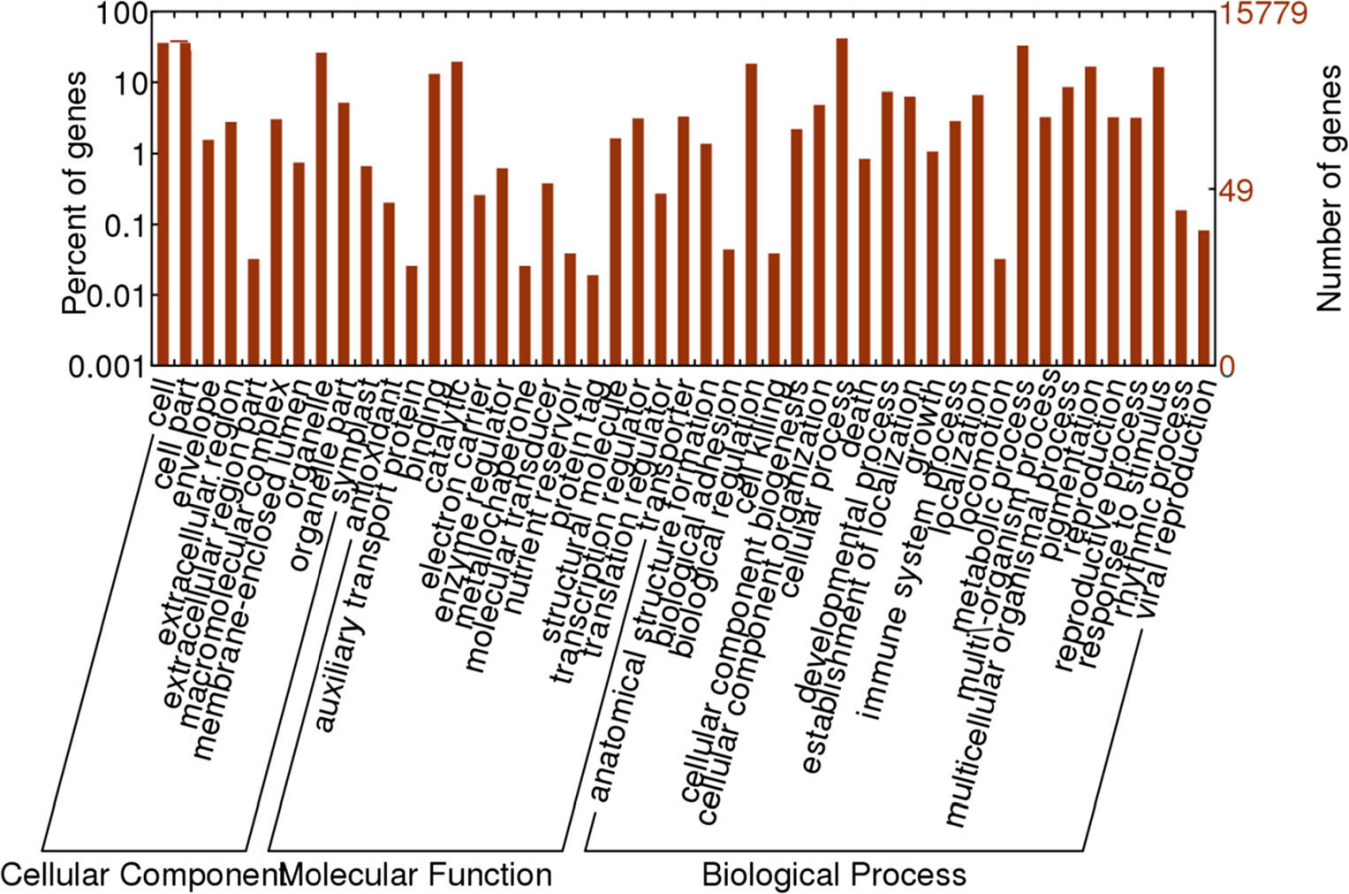
Gene ontology (GO) functional classification of 15,779 detected genes with annotated GO terms of *Brassica rapa*. The results are summarized in the three main categories of cellular component, molecular function and biological process, containing 10, 14 and 23 functional groups, respectively. Data are presented on log10 scales as percentages and actual numbers

In order to understand the gene expression and examine the macro-level distribution of gene functions for PEG-induced osmotic stress in both the DS and DT genotypes, we first retrieved the GO annotations of all upregulated genes from the GO database. Of the differentially expressed genes, 15,779 had assigned GO terms. The functional classifications of the GOs retrieved by the WEGO online software were classified into 10, 14 and 23 functional groups corresponding to the three GO output categories ‘cellular component’, ‘molecular function’ and ‘biological process’, respectively (Fig. 3).

**Fig. 4:**
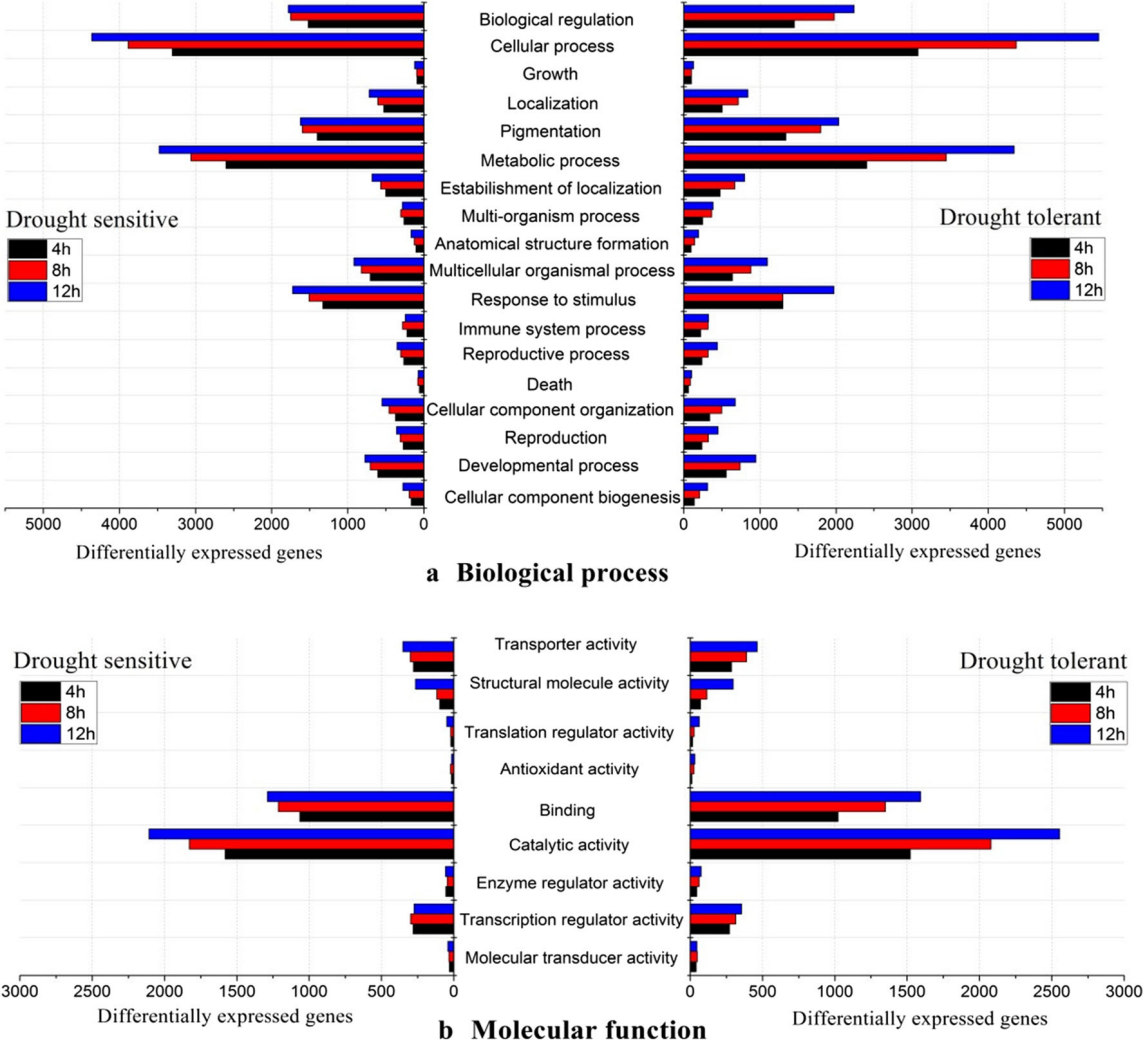
Functional groups of gene ontology (GO) categories of **a** biological process and **b** molecular function showing the numbers of gene functions assigned from differentially expressed genes in a drought-sensitive (DS) (*left side*) and drought-tolerant (DT) (*right side*) genotype of *Brassica rapa* at three times after initiation of treatments (4, 8 and 12 h)

The number of GO terms corresponding to the differentially expressed genes increased from each time point to the next in both the DS and DT accessions for the biological process categories ‘cellular process’, ‘metabolic process’ and ‘response to stimulus’ (Fig. 4a) and for the molecular function category ‘binding and catalytic activity’ (Fig. 4b). Notably, considerably more upregulated differentially expressed genes were assigned to the biological process ‘response to stimulus’ in DT than in DS, especially at 12 h.

### Enriched Biological Processes in Response to Osmotic Stress

GO enrichment analysis was applied to identify biological processes that were unique to the DT accession or common to both the DS and DT accessions. A list of significantly enriched GO terms from upregulated genes (*P* < 0.01) is shown in additional files (Table S1c–q). Upregulated GO terms in the PEG-treated vs. MS controls in both the DS and the DT accessions included ‘sulphur utilization’ (GO: 006791) and ‘regulation of sulphur utilization’ (GO: 006792) at all three time points (Tables S1c-e, Figs. **S1**a-c). At 4 h, ‘indole glucosinolate biosynthetic process’ (GO: 0009759) and ‘phosphorelay signal transduction system’ (GO: 0000160) were enriched in both DS and DT (Table S1c), while at 8 h, we also observed an enrichment of genes related to ‘peroxisome organization’ (GO: 0007031) and ‘cellular response to glucose stimulus’, a main source for H_2_O_2_ production, and genes involved in photosynthesis machinery and chloroplast organization (Table S1d). Finally at 12 h, both genotypes show a common response to the restricted water supply by the enrichment of genes belonging to the GO term ‘response to water stimulus’ (GO: 0009415). Additionally, at this time point, we see enrichment of genes related to ‘ethylene metabolism’ (GO: 0009692) (Tables S1e).

Unique to the DS accession, the upregulated genes at the first time point (4 h) were particularly enriched for GO terms such as ‘glucuronoxylan biosynthetic process’ (GO: 0010417), ‘regulation of phosphate metabolism’ (GO: 0019220), ‘fertilization’ (GO: 0009566), ‘autophagy’ (GO: 0006914), ‘DNA endoreplication’ (GO: 0042023) and ‘circadian rhythm’ (GO: 0007623) (Table S1f, Fig. **S1**d). Most of these metabolic processes are downstream of the ABA signalling cascade and have mainly a protective function against drought stress. ‘Glucuronoxylan biosynthetic process’ was observed again in the DS accession after 12 h exposure to the PEG, but not at any time point in the DT accession (Table S1h). No further enrichment for the response to osmotic stress was observed in the DS accession until 12 h after stress application (Tables S1g-h, Figs **S1**e, f).

In contrast, a much higher number of upregulated genes in the DT accession were annotated with GO-terms related to osmotic stress-related metabolism. A more sophisticated and complex regulatory response was activated in the DT accession just 4 h after the osmotic stress application, with genes related to the terms ‘systemic acquired resistance’ (GO: 0009627), ‘regulation of reactive oxygen species metabolism’ (GO: 2000377), ‘mitogen-activated protein kinase (MAPK) cascade’ (GO: 0000165), ‘response to salicylic acid’ (GO: 0009751), ‘regulation of cell death’ (GO: 0010941) and ‘cellular response to jasmonic acid stimulus’ (GO: 0071395) prominently represented (Table S1i, Fig. **S1**g). A similar complexity of the regulatory response was identified at all of the three time points (Tables S1i-k, Fig. **S1**g-i).

For genes upregulated uniquely in one of the two accessions, a similar trend was also observed for the GO terms of the category ‘molecular functions’. In this case, calcium-dependent protein kinases, calcium-related binding and heat shock proteins were enriched in the DT accession at 4 h and 8 h after imposition of stress (Tables S1o-q), while a similar trend was not detected in the DS accession until 12 h after stress application (Tables S1l-n).

**Fig. 5:**
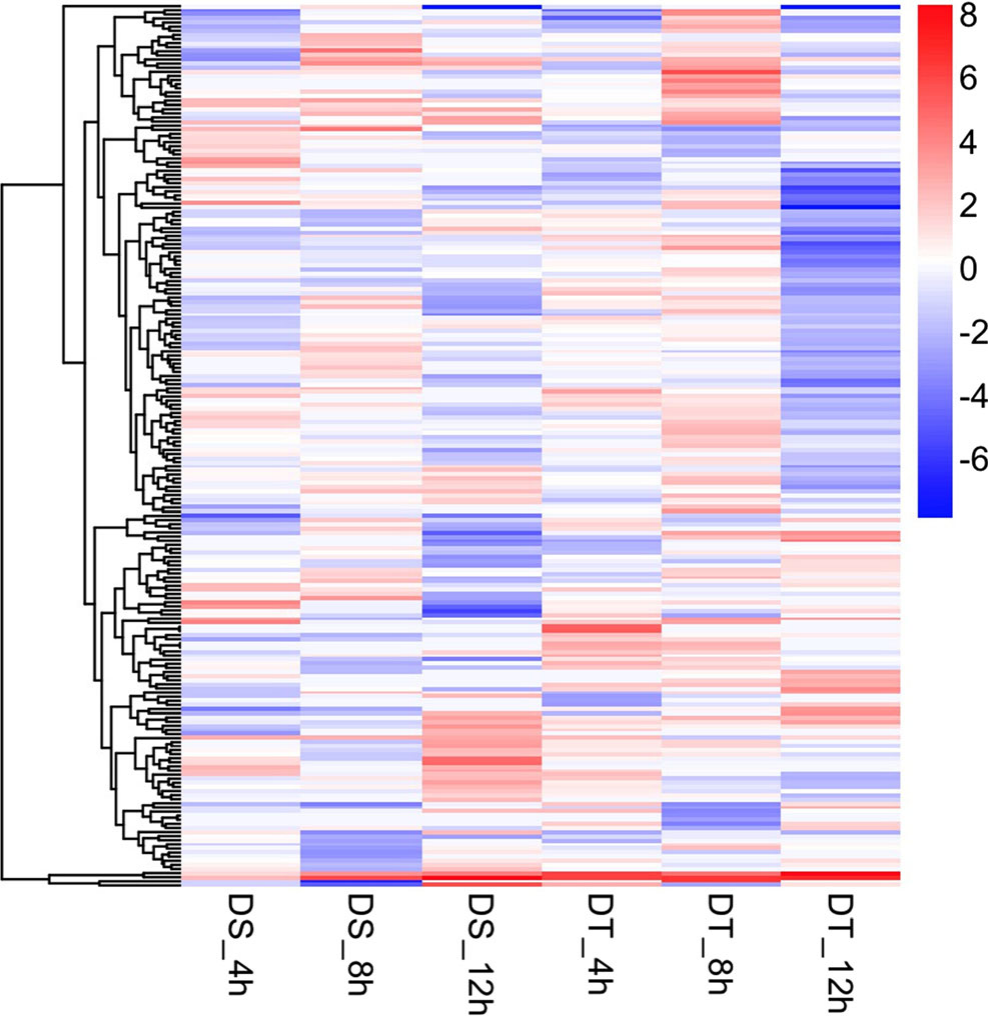
Selection of 215 drought-responsive genes in *Brassica rapa* from the gene ontology (GO) term ‘response to stimulus’. Values in the figure are FPKM change of PEG-stressed and MS control conditions in the drought-sensitive and drought-tolerant genotypes

A total of 215 genes with documented responsiveness to drought or osmotic stress were selected from the GO annotation ‘responsive to stimulus’ (GO: 0050896). Figure 5 shows a heat map presenting the log_2_-fold gene expression changes of these selected genes between PEG-stressed and MS control conditions in the DS and DT genotypes.

### Comparison of Gene Ontology Classification and Pathway Enrichment Analysis

Similar processes were identified in the DT and DS accession by GO classification and pathway enrichment analysis (MapMan) (Table S2). MapMan provides a general description of gene pathways, while GO specifies the pathways. It was normal for GO classification to identify several related pathways for every one pathway identified by MapMan (Table 2). By and large, the pathway enrichment analysis (MapMan) supported the GO classification analysis.

**Table 2:** Comparison between gene ontology enrichment analysis and pathway enrichment analysis (MapMan) for upregulated genes specific to the drought-tolerant (DT) genotype or common to both the DT and drought-sensitive (DS) genotypes of *Brassica rapa* 4 h after the beginning of the osmotic stress treatment

Gene ontology enrichment analysis		Pathway enrichment analysis (MapMan)
Uniquely upregulated in the DT genotype at 4 h		
Biological process	GO: 0012501 programmed cell death	cell.cycle
Biological process	GO: 0043067 regulation of programmed cell death	cell.cycle
Biological process	GO: 0009751 response to salicylic acid stimulus	hormone metabolism.jasmonate.induced-regulated-responsive-activated
Biological process	GO: 0000375 RNA splicing, via transesterification reactions	RNA.regulation of transcription.unclassified
Biological process	GO: 0000394 RNA splicing, via endonucleolytic cleavage and ligation	RNA.regulation of transcription.unclassified
Biological process	GO: 0000103 sulphate assimilation	S-assimilation.ATPS
Biological process	GO: 0002376 immune system process	stress.biotic
Biological process	GO: 0009627 systemic acquired resistance	stress.biotic
Biological process	GO: 0045087 innate immune response	stress.biotic
Biological process	GO: 0006955 immune response	stress.biotic
Biological process	GO: 0009814 defence response, incompatible interaction	stress.biotic
Biological process	GO: 0009607 response to biotic stimulus	stress.biotic
Molecular function	GO: 0003777 microtubule motor activity	cell.organisation
Molecular function	GO: 0008017 microtubule binding	cell.organisation
Molecular function	GO: 0005516 calmodulin binding	signalling.calcium
Molecular function	GO: 0005509 calcium ion binding	signalling.calcium
Molecular function	GO: 0004698 calcium-dependent protein kinase C activity	signalling.calcium
Upregulated genes common to both DT and DS genotypes at 4 h		
Biological process	GO: 0006791 sulphur utilization	S-assimilation.APR
Biological process	GO: 0006792 regulation of sulphur utilization	S-assimilation.APR
Biological process	GO: 0019419 sulphate reduction	S-assimilation.APR
Biological process	GO: 0010438 cellular response to sulphur starvation	S-assimilation.APR
Biological process	GO: 0061025 membrane fusion	protein.degradation.ubiquitin.E3.SCF.FBOX
Biological process	GO: 0006944 cellular membrane fusion	protein.degradation.ubiquitin.E3.SCF.FBOX
Biological process	GO: 0080163 regulation of protein serine/threonine phosphatase activity	amino acid metabolism.synthesis.serine-glycine-cysteine group.glycine.sarcosine oxidase
Biological process	GO: 0000079 regulation of cyclin-dependent protein serine/threonine kinase activity	amino acid metabolism.synthesis.serine-glycine-cysteine group.glycine.sarcosine oxidase
Biological process	GO: 0000160 phosphorelay signal transduction system	signalling.phosphorelay

## Discussion

This study provides the first detailed sequence-based analysis of transcriptomes from *B. rapa* accessions with tolerance or sensitivity to drought stress. We focussed on changes to transcriptomes during the first 12 h of osmotic stress in seedlings, compared to the gene expression under non-stress control conditions. Analysis of gene expression after stress application may improve our understanding of the mechanisms underlying the regulation of drought stress susceptibility and tolerance in this species. The results are highly relevant not only for *B. rapa*, which includes a variety of important vegetable, oilseed and fodder crops, but also for close relatives for which the *B. rapa* gene pool is frequently exploited to enrich breeding pools. In particular, *B. rapa* is an important source of genetic diversity for the gene pool of the globally important oilseed crop canola/oilseed rape (*B. napus*).

### Drought Tolerance Is Associated with Rapid Activation of Drought-Responsive Gene-Expression Networks

One of the most striking differences between the DS and DT accessions in their response to the applied osmotic stress was the speed of reaction in terms of expression of general active stress-responsive pathways. The initial response of the DS accession shows an enrichment of genes involved in the glucuronoxylan biosynthesis process, fertilization, autophagy, DNA endoreplication and circadian rhythm. This indicates that stress symptoms were being registered at this time point in the DS accession; however, little evidence was observed for the induction of a proactive response to combat the stress. The DT accession, on the other hand, showed a rapid and pronounced activation of multiple stress response mechanisms, including response networks to systemic acquired resistance, SA and MAPK cascade. Furthermore, drought-related transcription factors such as calcium-dependent protein kinase (CDPK) genes were upregulated in the DT accession 4 h after stress induction. A corresponding stress-related gene activation was not induced in the DS accession until 12 h after the imposition of the osmotic stress treatment. In general, the results from GO enrichment analysis were supported by pathway enrichment analysis (MapMan) (Table 1). The rapid and multifaceted stress response in the DT accession can be assumed to provide a considerably greater protection from water deficiency following the onset of the osmotic stress.

### Early Drought Responses Are Induced by Hormone Signalling and Regulation

Various signalling pathways are involved in the regulation of plant responses to osmotic stress. Enrichment for hormone-related regulation and signalling pathways in the DT accession 4 h after stress induction indicates that these pathways play an important role in the early stress tolerance response. The well-known stress response hormone SA generates a wide range of metabolic and physiological responses in relation to abiotic stress in plants (Hayat et al. 2010; Huang et al. 2008). In various crops such as rice, wheat and barley, drought tolerance has been found to be improved by applying exogenous SA (Farooq et al. 2009; Fayez and Bazaid 2014; Kang et al. 2013). Our results showed rapid and unique enrichment of genes involved in cellular response and signalling pathways related to SA and JA in the DT accession. In contrast, no enrichment of SA- or JA-related genes was observed in the DS accession until 12 h after stress imposition. The DS accession failed to respond to the onset of osmotic stress with an active physiological protection process (Fig. **S1**d-f).

The phytohormone SA can have a large impact on the regulation of drought responses. In *Phillyrea angustifolia*, the endogenous SA levels are increased up to fivefold (Munné-Bosch and Peñuelas 2003) and in barley roots around twofold (Bandurska and Ski 2005) after exposure to drought stress. The mechanisms underlying the influence of SA on drought tolerance are not clear especially as different concentrations show different effects. A low concentration of SA leads to a higher drought tolerance, whereas a high concentration tends to decrease the drought tolerance (Hamada and Al-Hakimi 2001; Kang et al. 2012; Korkmaz et al. 2007). SA is well known to induce systemic acquired resistance to pathogens (Hayat et al. 2010). Interestingly, there was enrichment of genes with the GO term ‘systemic acquired resistance’ in the DT accession at 4 h (Table S1i).

JA can influence stomatal closure. The treatment of *Arabidopsis* with the JA precursor 12-oxo-phytodienoic acid (12-OPDA) leads to the closure of the stomata. Additionally, increased OPDA levels can be correlated to increased drought tolerance. It was assumed that drought stress prevents the conversion of 12-OPDA to JA and OPDA acts independently or together with ABA on regulation of stomatal closure (Tatyana et al. 2014). JA may also be involved in modulating the root hydraulic conductivity which influences water uptake (Sánchez-Romera et al. 2014). In rice, the bHLH protein OsbHLH148, which leads to enhanced drought tolerance when it is overexpressed, interacts with rice jasmonate ZIM-domain (OsJAZ1) to activate the expression of *OsDREB1*, which serves as a marker for drought responses (Seo et al. 2011).

### Early Response of DS Plants to Drought Stress Focuses on a Prevention of Cell Damage

After 4 h osmotic stress, genes involved in autophagy, DNA endoreplication, circadian rhythm, fertilization and glucuronoxylan biosynthesis process were enriched in the DT accession. The DS plants reacted in a passive manner by preventing cell damage and focusing on ‘survival of the species’ by expressing genes involved in the reproductive process (Fig. **S1**d-f).

### Early Drought Adaption Is Induced by Abscisic Acid Signalling in the DS and DT Genotypes

ABA is a key phytohormone in the early response to drought stress (Cutler et al. 2010). ABA is synthesized in the roots upon sensing of limited water conditions in the soil and transports the signal through the xylem from the roots to the shoots (Hartung et al. 2002; Jiang and Hartung 2008). There it induces an H_2_O_2_-dependent closure of the stomata to reduce transpiration-mediated water loss (Sharma and Verslues 2010).

In this study, both genotypes showed adaptive processes that were induced by the ABA signalling cascade. In the DS genotype, genes involved in autophagy, fertilization and circadian rhythm were enriched at 4 h (Table S1f); in the DT genotype at the same time point, there was an enrichment of genes involved in PCD, and genes enriched for the GO term ‘responsive to biotic stimulus’ including genes relevant for the activation of the ABA-mediated signalling and regulation pathway (Table 1). At 8 h, in both genotypes, the significantly enriched genes include ‘peroxisome organization’ and the ‘cellular response to glucose stimulus’ (Table S1d).

Autophagy is a mechanism that removes toxic or damaged cellular components and recycles nutrients. It is initiated in the cytoplasm by forming membranes that enclose these molecules, making so-called autophagosomes, which then fuse with the vacuoles to release their contents. ABA induces autophagy indirectly as the ABA treatment leads to the generation of oxidized proteins and potentially damaged organelles which are removed by autophagy. In the DS genotype the expression cascade triggered by the putative ABA response appears to be related to autophagy and circadian rhythms, indicating a focus on prevention of cell damage or passive stress avoidance. Under drought stress, ABA is also known to be transported to the plant reproductive structures and to influence the development of plants by expression changes in genes controlling cell division (Barnabas et al. 2008). The activation of fertilization genes observed in the DS genotype (Fig. **S1**d-f) is consistent with a mobilization of resources to achieve successful reproduction before the onset of further stress.

Also, the carbohydrate metabolic enzyme activity can be influenced by ABA (Liu et al. 2005), which is consistent with our study showing an enrichment of genes that respond to glucose stimulus in both accessions at 8 h. Evidence suggests that there is an overlap of genes relevant for controlling the circadian clock and ABA-dependent regulation (Matsui et al. 2008; Takeshi and Takafumi 2008) in response to drought and water deprivation. Additionally, key enzymes in ABA precursors and biosynthesis are controlled by the circadian clock (Takeshi and Takafumi 2008).

PCD affords cells the ability to self-destruct in a controlled manner as a response to stress. PCD is believed to be regulated and involved with plant development and plant defence (van Doorn and Woltering 2004), and is a well-known resistance response to attacks by biotrophic pathogens or the death of plant cells in compatible host-parasite interactions (Li et al. 2008; Lincoln et al. 2002). Like hormone signalling and other stress-responsive genes, PCD genes can also be induced by various abiotic stimuli such as salt stress, osmotic stress or endoplasmic reticulum stress (Cai et al. 2014; Duan et al. 2010; van Doorn and Woltering 2004; Wang et al. 2010), particularly in plant roots. An endoplasmic-reticulum stress-induced PCD was observed in Arabidopsis seedlings when they were treated with increasing concentration of PEG 8000 (Cai et al. 2014). Under water-deficient situations, ABA and reactive oxygen species induce senescence in plant leaves and root tips, executed by PCD. This mechanism plays an important role for the plant in survival as it remobilizes nutrients during stress allowing the rest of the plant to survive. An additional function of PCD is the abscission of leaves to reduce water loss through transpiration (Duan et al. 2010).

### Differential Transcription Factor Activation and Signal Responses in the Osmotic Stress Reaction

Transcription factors play critical roles in the modulation of gene expression in response to drought and other abiotic stresses. Calcium-related transcription factors, such as CDPKs, calcium B-like proteins and calcium-modulated proteins (calmodulin—CaM), along with their corresponding kinases (CIPKs), were frequently reported to regulate resistance responses against abiotic stress. For example, Pandey et al. (2013) demonstrated that the calmodulin transcription activator *CAMTA 1* regulates drought responses in *A. thaliana*.

Corresponding to this finding, we observed a clear enrichment of genes involved in calcium-related binding or signalling in the DT accession at 4 and 8 h after stress application (Table S1 o-q). In contrast, the DS accession was characterized by a much more passive regulatory response throughout the duration of the stress treatment (Table S1 l-n). Transcriptional activators of calcium-modulated stress responses therefore also appear to be highly interesting candidates for regulation of drought resistance strategies in *Brassica* species.

### Sulphur Assimilation and Metabolism Play Important Roles in Drought Response in *B. rapa*

Genes involved in sulphur assimilation and regulation were differentially expressed in both the DS and the DT accession throughout the entire duration of the stress treatment; this observation was common to the GO analysis and the pathway analysis. Sulphur is an essential element for plant growth due to its presence in proteins, glutathione, phytochelatins, thioredoxins, chloroplast membrane lipids, and certain coenzymes and vitamins (Khan et al. 2010). Sulphur applied as sulphate or the element sulphur has been found to play a significant role in drought stress signalling and responses (Chan et al. 2013).

Different regulators of sulphur metabolism have been identified, but the signal transduction towards supply and demand, as well as coordination with other assimilatory pathways, is unknown (Takahashi et al. 2011). The observed changes in sulphur regulation could be caused by a regulatory function of ABA signalling in sulphate metabolism, as ABA regulates several components of sulphur assimilation including sulphate transport, cysteine synthesis and flux into primary assimilation. On the other hand, there is as yet no clear understanding of these processes (Urano et al. 2009), making this topic of great interest for future exploration.

Similarly, glucosinolate biosynthetic and metabolic processes also showed stress responsiveness in both accessions at 4 h. Glucosinolates are a class of secondary metabolites with high sulphur content that exist exclusively in Brassicaceae. Besides a clear interactive role with regard to both beneficial and harmful biotic factors, glucosinolates may also play a role in regulation of drought stress (del Carmen et al. 2013). Interestingly, as glucosinolates may represent up to 30% of the total sulphur content of plant organs, the regulation of glucosinolates is intimately related to the sulphur status of the entire plant (Falk et al. 2007). An increased sulphur supply has been shown to result in higher levels of total glucosinolates in *Brassica* species after moderate or severe drought stress (Gutbrodt et al. 2012; Schreiner et al. 2009; Zhang et al. 2008). However, when sulphur supply is deficient, there is a general downregulation of glucosinolate biosynthetic genes, accompanied by an upregulation of genes controlling sulphur uptake and assimilation (Falk et al. 2007). A similar response was clearly observed in our study: glucosinolate biosynthetic process genes were quickly upregulated only 4 h after osmotic stress application in both accessions (Table S1c).

### Drought Stress Tolerance Is Modelled by Changes in the Glucuronoxylan Biosynthesis

Glucuronoxylan is one of the major components of the secondary cell walls. In Arabidopsis, mutations in genes from the glucuronoxylan pathway were shown to modulate drought tolerance, e.g. a T-DNA insertion mutant for irx14, a glycosyl transferase, results in a drought-tolerant phenotype (Keppler and Showalter 2010); a mutation in the *CesA8/IRX1* gene (*lew2*), which encodes a subunit of a cellulose synthesis complex, leads to the increase of osmolytes, such as sugar and proline, which increase drought and osmotic tolerance and acts as a protection against drought stress (Chen et al. 2005). In our study, the DS genotype showed an enrichment of upregulated genes belonging to the GO term glucuronoxylan biosynthesis process at 4 and 12 h, but not in the DT genotype (Table S1f-h). The two genotypes show different gene expression patterns within the glucuronoxylan biosynthesis pathway that might influence their tolerance to drought stress.

### Validation of RNA-Seq Data by qRT-PCR

RNA-Seq gene expression was validated by qRT-PCR in several stress-related genes in drought-affected DT and DS *B. rapa* at first flower. The genes and gene pathways stimulated by osmotic stress that we report here by RNA-Seq require further validation under drought-stress conditions.

### *B. rapa* as a Source of Genetic Variation for Improvement of Drought Tolerance in *B. napus*

Hatzig et al. (2014) applied a similar osmotic treatment system to investigate hormone metabolism and metabolic status in DT and DS *B. napus* genotypes. That study also revealed a similar hormonal adjustment and osmolyte accumulation in DT genotypes compared to DS genotypes; however, the differentiation of the stress response between tolerant and susceptible *B. napus* genotypes did not occur until considerably later, when the stress treatment (PEG concentration) was increased*.* Furthermore, the main difference between the DT and DS *B. napus* genotypes seemed to be characterized by a lack of response of the DS genotype compared to the DT genotype, similar to what we observed in *B. rapa* in this study. We show an extremely rapid activation of multiple, proactive stress defence mechanisms in DT *B. rapa*, suggesting that DT *B. rapa* may harbour a superior, rapid stress resistance modulated by rapid induction of multiple, complex regulatory stress response pathways. We conclude that genes in the DT *B. rapa* genotype, induced at a very early stage after the onset of stress, lead to a rapid cascade of molecular and biological changes that confer different layers of morphological and metabolic protection against drought.

## Conclusions

Gene ontology enrichment analysis identified biological processes and metabolic pathways that differentiated the response of DS and DT genotypes of *B. rapa* to simulated drought stress. In the drought-sensitive genotype, the expression cascade triggered by the putative ABA response appears to be related to autophagy and circadian rhythm, indicating a focus on prevention of cell damage or passive stress avoidance. A specific and complex regulatory response was activated in the DT genotype at 4 h after the osmotic stress application, including response networks to systemic acquired resistance and PCD. The ABA-induced expression of genes related to PCD indicates an active strategy to maintain normal metabolism. In comparison to *B. napus* (canola), *B. rapa* may harbour a superior and more rapid response to drought stress. Subsequent research should focus on validation of these results through independent tests of gene expression (such as reverse-transcriptase quantitative PCR) and the development of biomarkers for selection of breeding materials with potentially improved drought tolerance and for transfer of drought tolerance from *B. rapa* to *B. napus*.

## Electronic Supplementary Material

Fig. S1. Significantly enriched gene ontology (GO) terms from the category ’biological process’ for 1) up-regulated genes in the PEG treatment relative to the control treatment commonly identified in the *Brassica rapa* drought-sensitive and drought-tolerant genotypes at a) 4 h, b) 8 h and c) 12 h time point, 2) up-regulated genes in the PEG treatment relative to the control treatment uniquely identified in the drought-sensitive (DS) genotype at the d) 4 h, e) 8 h and f) 12 h time point and 3) up-regulated genes in the PEG treatment relative to the control treatment uniquely identified in the drought-tolerant (DT) genotype at the g) 4 h, h) 8 h and i) 12 h time point. The web-based tool REViGO (http://revigo.irb.hr) was used to reduce the redundancy of the GO terms and visualize the reduced lists at the three time points. Each disc represents a GO term. The disc size is proportional to the frequency of this GO term in the underlying Gene Ontology Annotation Database (discs of more general terms are larger; discs of more specific terms are smaller) (Supek et al. 2011). Spatial arrangement of discs approximately reflects a grouping of GO categories by semantic similarity. The scatterplots show the cluster representatives (i.e. terms remaining after the redundancy reduction) in 2-D space. (DOCX 755 kb)

Table S1 (XLSX 3733 kb)

Table S2 (XLSX 27 kb)

Table S3 (DOCX 22 kb)
