# Supplementary figures and images for: Drought-Tolerant Brassica rapa Shows Rapid Expression of Gene Networks for General Stress Responses and Programmed Cell Death Under Simulated Drought Stress
Source: Plant Mol Biol Report. 2017 May 22;35(4):416–30. doi: 10.1007/s11105-017-1032-4 (PMC5504209; doi:10.1007/s11105-017-1032-4)

**Supp. Fig. 1**

a)


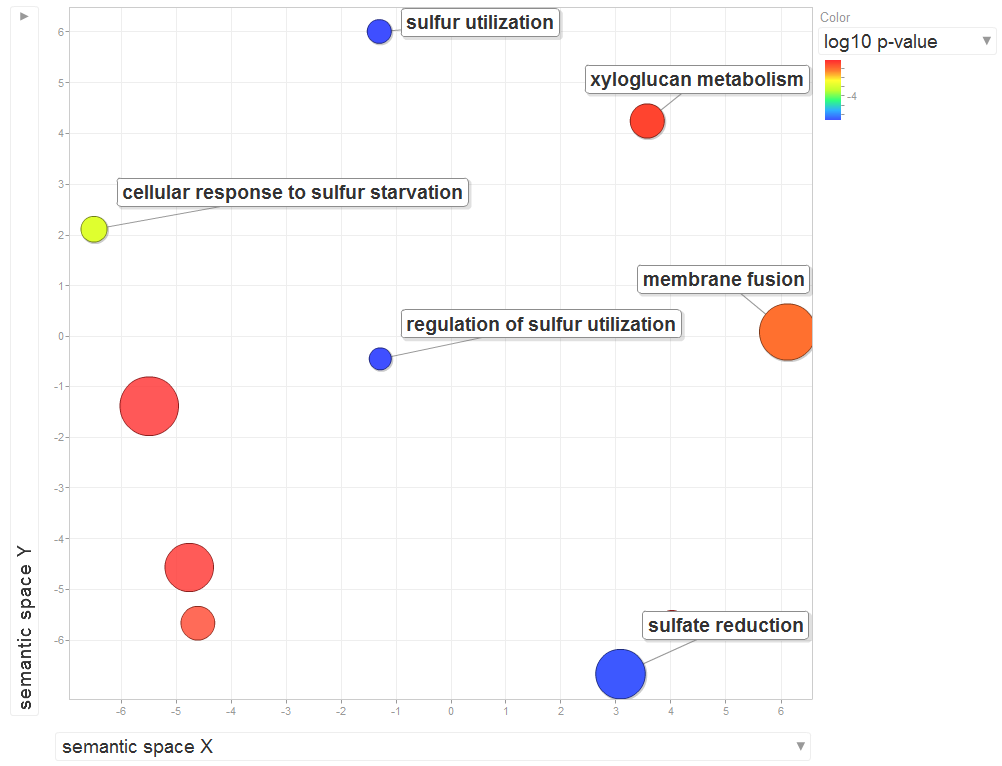


b)


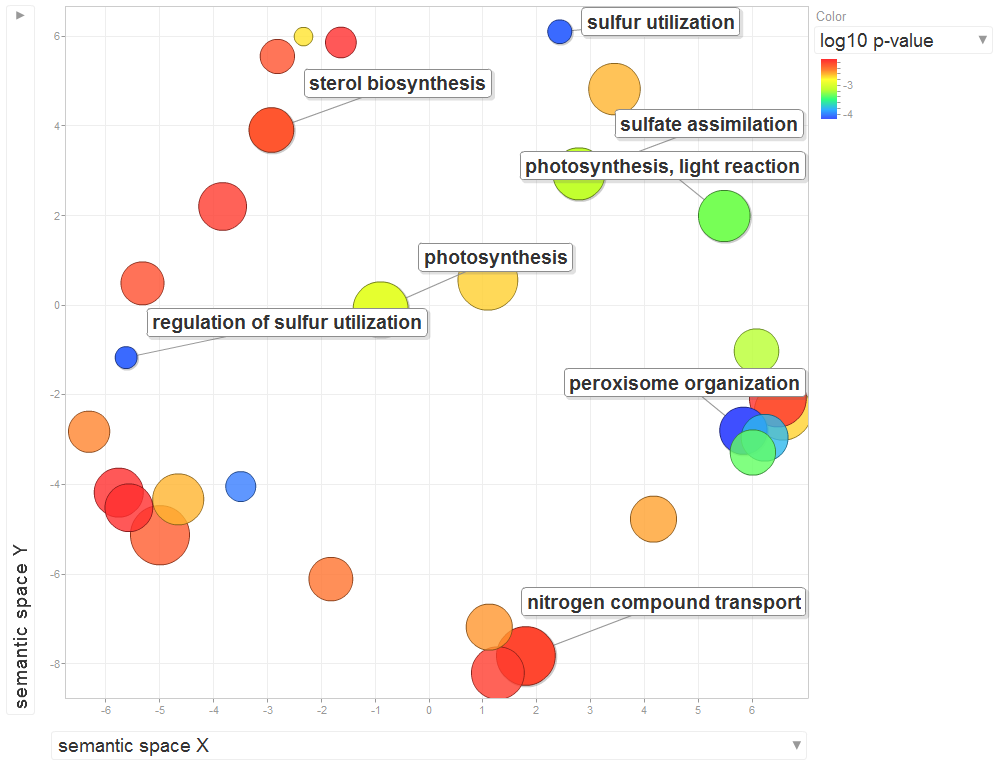


c)


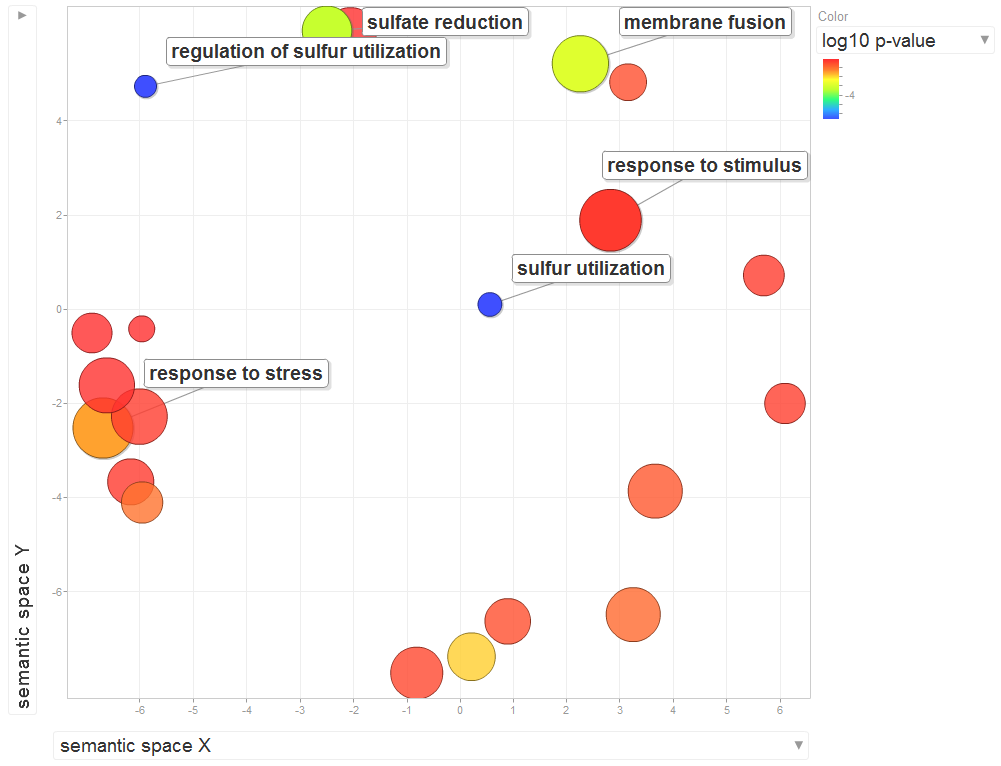


d)


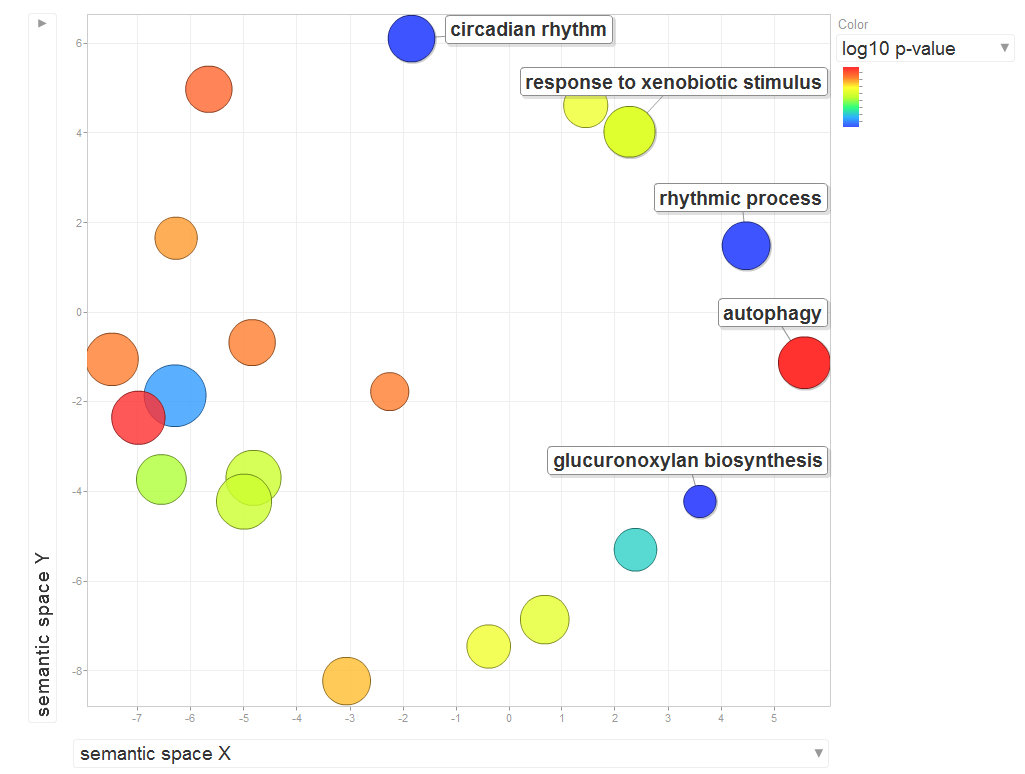


e)


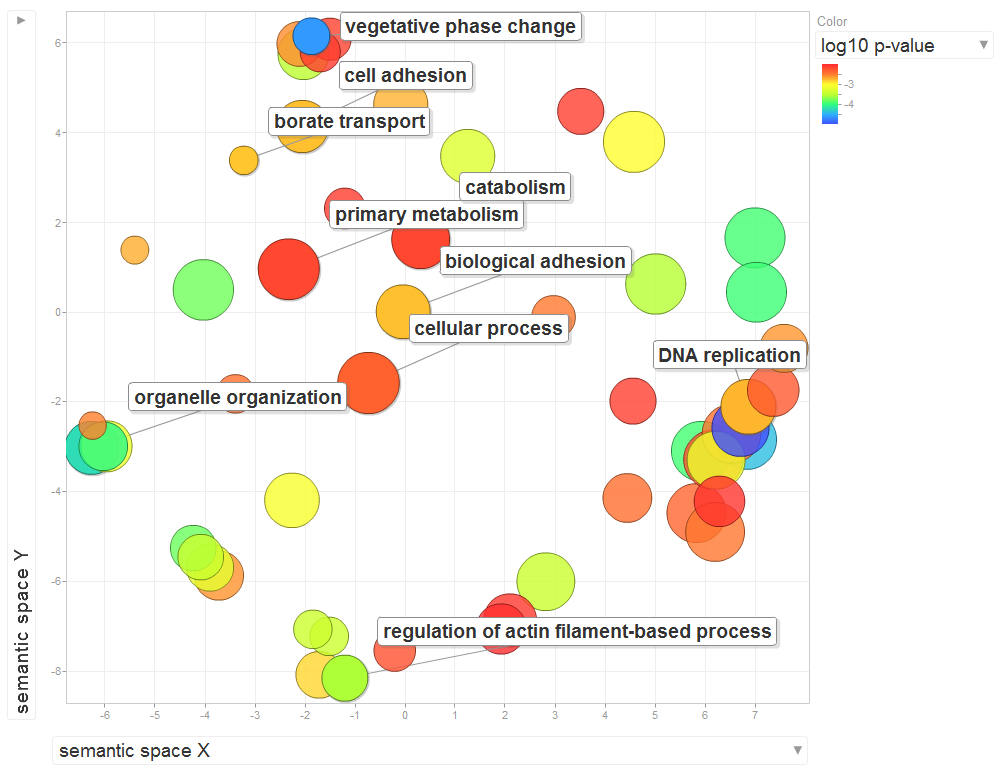


f)


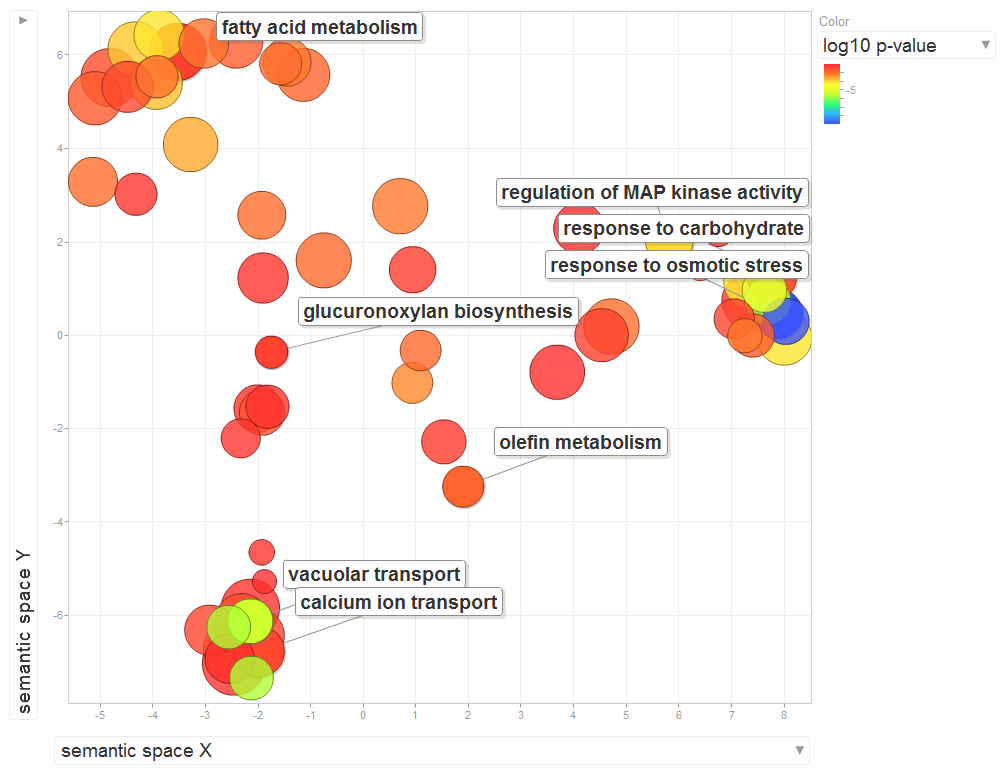


g)


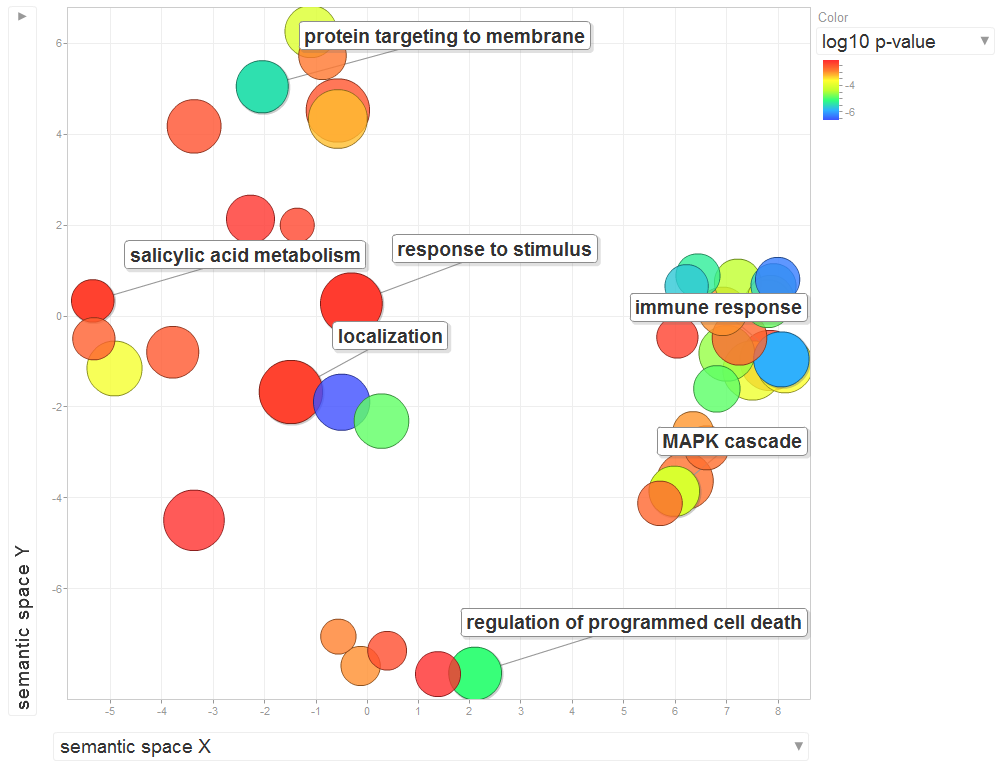


h)


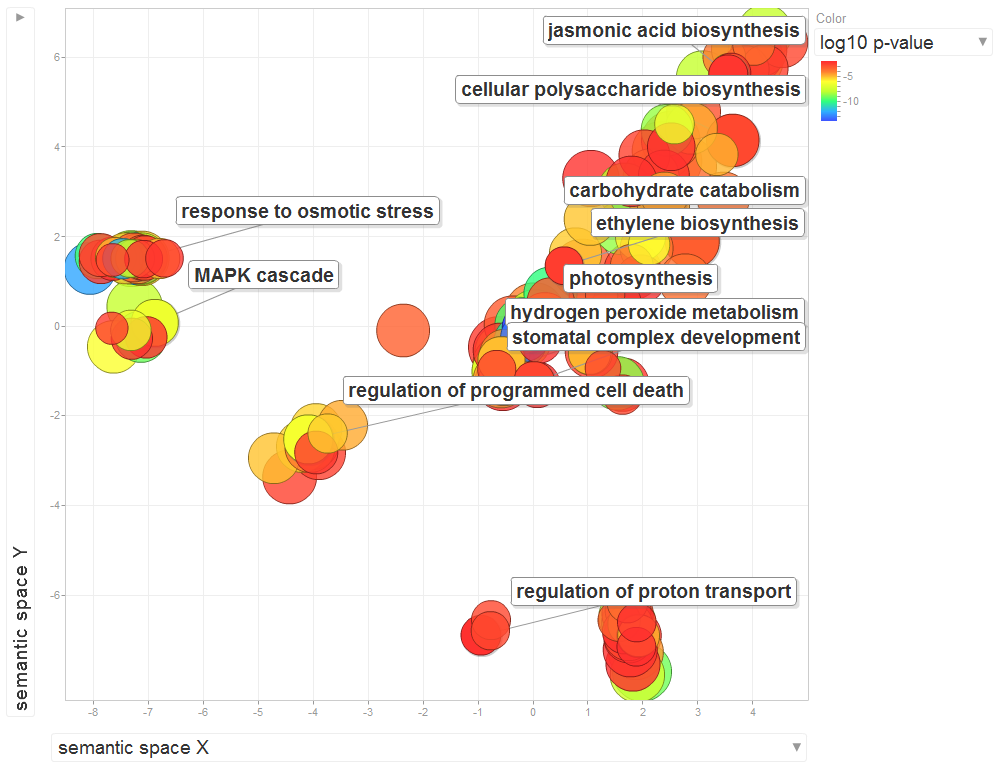


i)


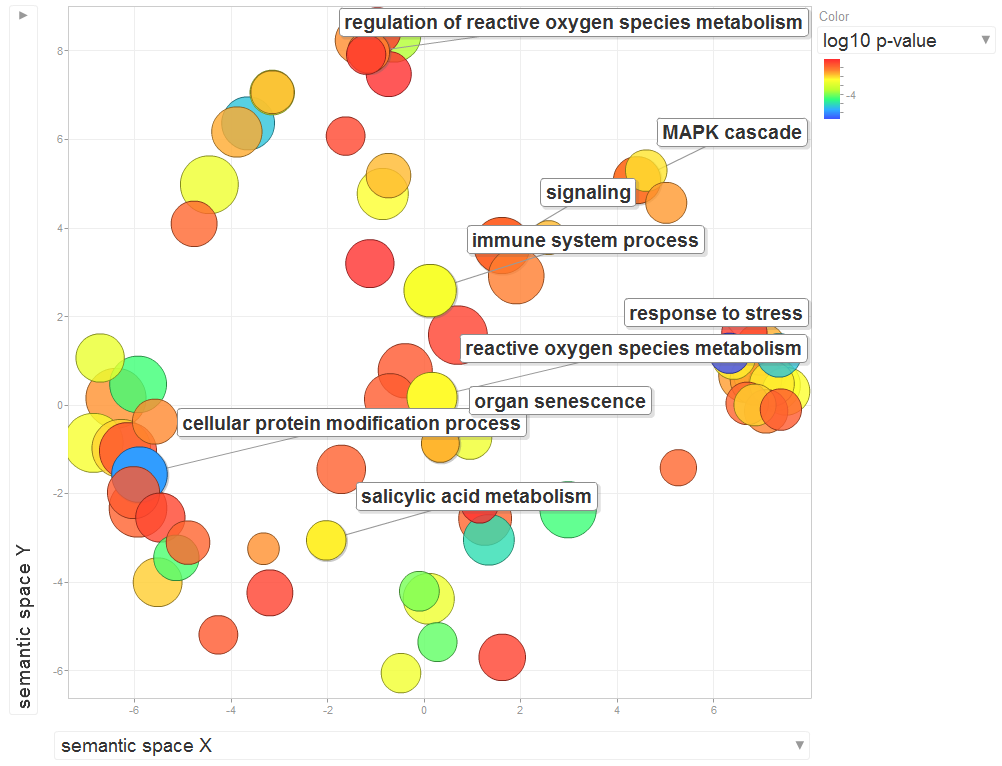

Supplement: Supplementary file 1 — Significantly enriched gene ontology (GO) terms from the category ’biological process’ for 1) up-regulated genes in the PEG treatment relative to the control treatment commonly identified in the Brassica rapa drought-sensitive and drought-tolerant genotypes at a) 4 h, b) 8 h and c) 12 h time point, 2) up-regulated genes in the PEG treatment relative to the control treatment uniquely identified in the drought-sensitive (DS) genotype at the d) 4 h, e) 8 h and f) 12 h time point and 3) up-regulated genes in the PEG treatment relative to the control treatment uniquely identified in the drought-tolerant (DT) genotype at the g) 4 h, h) 8 h and i) 12 h time point. The web-based tool REViGO (http://revigo.irb.hr) was used to reduce the redundancy of the GO terms and visualize the reduced lists at the three time points. Each disc represents a GO term. The disc size is proportional to the frequency of this GO term in the underlying Gene Ontology Annotation Database (discs of more general terms are larger; discs of more specific terms are smaller) (Supek et al. 2011). Spatial arrangement of discs approximately reflects a grouping of GO categories by semantic similarity. The scatterplots show the cluster representatives (i.e. terms remaining after the redundancy reduction) in 2-D space. (DOCX 755 kb) [file 11105_2017_1032_MOESM1_ESM.docx]
