# Supplementary material for: Drought-Tolerant Brassica rapa Shows Rapid Expression of Gene Networks for General Stress Responses and Programmed Cell Death Under Simulated Drought Stress
Source: Plant Mol Biol Report. 2017 May 22;35(4):416–30. doi: 10.1007/s11105-017-1032-4 (PMC5504209; doi:10.1007/s11105-017-1032-4)
Supplement: Supplementary file 4 — (DOCX 22 kb) [file 11105_2017_1032_MOESM4_ESM.docx]

Table S3. List of 29 candidate genes and their primer sequences for qRT-PCR validation.

| No | Gene ID | Name | Function | Forward Primer (5' to 3') | Reverse Primer (5' to 3') | Expected PCR  Product (bp) |
| --- | --- | --- | --- | --- | --- | --- |
| 1 | Bra000703 | HSP22 | BrHSP22 | AGCGTGGCTCTGTCTCCA | CTTCCTCTCTTTTCCTCTCTCCA | 157 |
| 2 | Bra005099 | LRP1 | Lipid transfer protein LTP1 | CTTGCCGTTGCCTTGTAGGA | GCAGTTGGTGGTTTTGCTGA | 122 |
| 3 | Bra005514 | HSP60 | BrHSP60-2 | TGACAACGACGGAAGCAGT | TCCCATACCGCCCATACCT | 110 |
| 4 | Bra006721 | PR14 | Pathogenesis-related PR-14 | TGCCTTGTATTGACGGTGTG | CGGTCTGGTGTGGTTTGAG | 176 |
| 5 | Bra007100 | PIP2;5 | Aquaporin PIP2;5 | AGGAAGTGGTTGGGGAGAAG | AGAGGAAGAGGAGAGTGGCTATG | 147 |
| 6 | Bra008661 | KIN2 | Cold-regulated protein COR6.6 (KIN2) | TGCTGCTACCTCTGCTGGA | CGGTCTTCTCCTTCACAACATT | 95 |
| 7 | Bra009225 | LEA.2 | Late embryogenesis abundant BnLEA4-1 | GATGTCGGCTTTGCCTGGT | GGTGTTATGAGCGGTGGTCCT | 121 |
| 8 | Bra010498 | Hsp101 | HSP101/ HSP100 | CGAGGAGAACCAAGAACAACC | TTAGCACCAGCGACCAAAG | 166 |
| 9 | Bra012696 | HSP70.2 | HSP70.2 KBFL-003C05 | TGCTGCCCTTGTCTATTACTCTG | CGCATCTCCATACTCTGTCCTC | 126 |
| 10 | Bra012883 | TF | Transcription factor-like | GGTAATCCCATCAAGCCAACAC | CCGTCGTGGTTCTTCTCTCTTC | 190 |
| 11 | Bra013206 | WSIP1 | Water stress-induced protein (WSIP1) | TCAACAAGATCGGTGATGCTCT | ATGTTTGTGGTCTCCGCTGCT | 182 |
| 12 | Bra013799 | CIPK1 | CBL-interacting protein kinase (CIPK1) | CACCATCCTCAAACGCAAG | CGTAGAGACGAACAACACAAGG | 92 |
| 13 | Bra014602 | UBL1 | Ubiquitin-protein ligase (UBL1) | GGTCTGTGAATCTAATGGTGTGG | TTGGAGGCTTGCTCGGATAG | 120 |
| 14 | Bra016073 | HS1 | Kuntiz type protease BocHS 1 | TATGGGCAGTCGATGTTTCC | CCTGGGATGGTTCCAACG | 160 |
| 15 | Bra016934 | ERD15 | ERD15 protein (ERD15) | AGGGAAGAACGGTGAGATGG | GCTGGTGAATGTTTCGTGGAG | 134 |
| 16 | Bra018216 | HSP17.4 | sHSP17.4 | GTGGAGAGAGAAGCAGCGAGA | ACCGTGACCGACAACACAC | 157 |
| 17 | Bra022955 | ARP.2 | Auxin-repressed protein (ARP1) | AAGGTGTAGGAGAAGGGAGCA | GGGAGCGTTTGGGTGAGAG | 188 |
| 18 | Bra023811 | PKL1 | Protein kinase-like protein (PKL1) | CGGAGATGGAGGAAGTCGTG | CGAAGCAGAAACAGCCAGGT | 99 |
| 19 | Bra026044 | ARP.1 | Auxin-responsive family protein (ARFP1) | GGCGAAGAGATGGAGAGGT | CGCTCAAACACGATGACG | 146 |
| 20 | Bra027181 | TIP2.1 | Aquaporin TIP2;1 | CGCTGTCGGTGGTCAAATC | CTCCTTCTATCGCTCCTACTCCA | 158 |
| 21 | Bra028875 | MP1 | Metallothionein protein (MP1) | TGCTGTGGAGGAAACTGTAGC | GCAACGCCGAGGACAAGA | 128 |
| 22 | Bra030858 | CPN60B | BrCPN60B | ATTGTCGTTGGTGGTGGTTG | CGTTGACTCCTGCGTTCTTG | 160 |
| 23 | Bra031127 | DNAJ1 | J-protein/HSP40 family DnaJ/Ydj1)) | CCGAGTTCTTTGGCTTCTCC | TCCTCCTCCTCCTGCTTCAC | 119 |
| 24 | Bra032742 | DNAJ2 | J-protein/HSP40 family (DnaJ/Ydj1) | TACCCGCTTCTTTCCCTTCT | TCCTCCGATTGCTTTACTGG | 112 |
| 25 | Bra032937 | TIP1;2 | Aquaporin TIP1;2 | CGCTTGCTTCCTCCTTCAGTT | GGCGATGGGTGCGATAGTT | 187 |
| 26 | Bra034845 | PP2C.2 | Putative protein phosphatase PP2C | CGAGAAGTTAGCCAGCGAGA | CAAAGCACGGCACGAGAG | 182 |
| 27 | Bra038734 | HSP70 | HSP70 | CGGGTAAAGGCGAAGGTC | GAAGGAGTGGTGCGGTTG | 124 |
| 28 | Bra040530 | PP2C.3 | Putative protein phosphatase 2C 34 | TCGTCTGGGAGGGATTTGG | CTGGTCCGTTTCGGGTTCT | 179 |
| 29 | Bra040968 | HSFB-2B | HSFB-2b/ HSF | CGCTGTCTCTCCGTCCAACTC | CACCAACCTGTCCTCCTCCTC | 101 |
